# Disease Severity and Mortality Can Be Independently Regulated in a Mouse Model of Experimental Graft versus Host Disease

**DOI:** 10.1371/journal.pone.0118079

**Published:** 2015-02-02

**Authors:** Rômulo G. Galvani, Ramon Lemos, Rômulo B. Areal, Pollyanna A. Salvador, Dario S. Zamboni, João Luiz M. Wanderley, Adriana Bonomo

**Affiliations:** 1 Divisão de Medicina Experimental, Coordenação de Pesquisa, Instituto Nacional de Câncer, Rio de Janeiro, Brazil; 2 Departamento de Imunologia, Instituto de Microbiologia Professor Paulo de Góes, Universidade Federal do Rio de Janeiro, Rio de Janeiro, Brazil; 3 Departamento de Biologia Celular, Molecular e Bioagentes Patogênicos, Faculdade de Medicina de Ribeirão Preto, Universidade de São Paulo, Ribeirão Preto, Brazil; 4 NUPEM, Campus Macaé Professor Aloísio Teixeira, Universidade Federal do Rio de Janeiro, Macaé, Rio de Janeiro, Brazil; 5 Laboratorio de Pesquisa sobre o Timo, Instituo Oswaldo Cruz, FIOCRUZ, Rio de Janeiro, Brazil; Beth Israel Deaconess Medical Center, Harvard Medical School, UNITED STATES

## Abstract

Graft versus host disease (GVHD) is the major limitation of allogeneic hematopoietic stem cell transplantation (HSCT) presenting high mortality and morbidity rates. However, the exact cause of death is not completely understood and does not correlate with specific clinical and histological parameters of disease. Here we show, by using a semi-allogeneic mouse model of GVHD, that mortality and morbidity can be experimentally separated. We injected bone marrow-derived dendritic cells (BMDC) from NOD2/CARD15-deficient donors into semi-allogeneic irradiated chimaeras and observed that recipients were protected from death. However, no protection was observed regarding clinical or pathological scores up to 20 days after transplantation. Protection from death was associated with decreased bacterial translocation, faster hematologic recovery and epithelial integrity maintenance despite mononuclear infiltration at day 20 post-GVHD induction with no skew towards different T helper phenotypes. The protected mice recovered from aGVHD and progressively reached scores compatible with healthy animals. Altogether, our data indicate that severity and mortality can be separate events providing a model to study transplant-related mortality.

## INTRODUCTION

Bone marrow transplantation (BMT) is a therapeutic strategy employed to treat malignant and non-malignant hematological diseases and primary immunodeficiencies. BMT envisages reestablishment of normal hematopoiesis and the therapeutic graft versus tumor effect. Graft versus host disease (GVHD) [1], a frequent complication post-BMT [2], may be responsible for 50% of the deaths in non-relapse patients [3]. However, the exact cause of death is not completely understood and frequently does not correlate with specific clinical and histological parameters of disease. For example, the most severe form of cutaneous acute GVHD (aGVHD) indicates a poor prognosis with very high mortality rates, but the cause of death is unrelated to the cutaneous disease [4]. Moreover, in a retrospective study it was shown that from 41% BMT patients who died from respiratory failure due to pulmonary hemorrhage, only 59% had significant aGVHD with pulmonary infiltrate [5]. Hypovolemic shock syndrome induced by TNFα-dependent systemic endothelial activation is related to GVHD mortality, in a mechanism similar to what occurs in sepsis [6]. Although TNFα serum levels are high in experimental models and in patients undergoing aGVHD [7–9], treatment with neutralizing anti-TNFα antibodies [10] confers about 50% protection from death in experimental models but has shown questionable results in human patients [7,8,11,12].

Several cellular interactions between donor/patient cells after transplantation can modulate disease. Both donor and recipient B cells, dendritic cells (DC), granulocytes, NK cells, myeloid-derived suppressor cells and regulatory T cells may play protective or pathogenic roles depending on the conditioning regimen, kinetics of cell administration and cell activation/differentiation status [13–15]. Regarding DC, either host or donor DCs can induce CD4^+^ T cell-dependent aGVHD whereas host APCs are required for CD8^+^ T cell-dependent disease [16–18]. Radiation-resistant host epidermal Langerhans and dermal dendritic cells become activated due to the inflammatory response following the conditioning regimen and are the main inducers of alloreactive T cell priming [19,20]. Although it has also been shown that the effector phase of acute GVHD can occur in the absence of MHC in the target tissue [21]. Adoptive transfer of plasmacytoid DCs can induce aGVHD in transplanted MHC-class II-deficient mice, depending on establishment of inflammation [22]. Prevention or treatment of GVHD can be achieved by either deletion or functional modulation of DCs [23].

The relationship of commensal microorganisms and development of aGVHD has been proposed almost 40 years ago [24,25] and confirmed in humans later on [26]. Thereafter, intestinal decontamination became a common practice in BMT [26–30] especially when the risk of GVHD development is high as in matched unrelated transplants or in related but not fully matched HLA [31]. With the knowledge about the pattern recognition receptors (PRR) in innate immune cells [32,33] and its subsequent role in the activation of DCs and consequently of lymphocytes [34], several authors have studied the role of PRRs in aGVHD development [35–43]. It was suggested [44] that the host milieu, submitted to the conditioning regimen, is activated by commensal microorganisms in such a way that donor T cells find the adequate environment within the host to be activated and trigger disease. This was corroborated by other findings showing not only that decontamination could diminish disease but that treatment with probiotics could also protect mice from aGVHD [45]. In humans, studies on the impact of the innate immune receptors small nucleotide polymorphisms (SNPs) on GHVD suggest a role for TLR4 and 9 in the outcome after BMT, but whether the effect is either related to the anti-graft reaction or to infections is not clear. Polymorphisms of the NOD2/CARD15 cytoplasmic receptor have been suggested as a high-risk variable for the development of aGVHD in 4 studies [35,37,38,43], when both donor and recipient carry the polymorphism. On the same line, other 3 studies show no differences in aGVHD occurrence in the presence of any NOD2 SNPs [36,40,42]. In one study however, the presence of variant forms of NOD2 in the donor cells promote protection, limiting the incidence of severe aGVHD to zero [35]. In the experimental setting, there is one study showing that NOD2 expression in the recipient hematopoietic cells is important to protect from aGVHD [41]. In addition, NOD2 molecules are considered important regulators of microbial-dependent inflammation, regulating NFkB activation and subsequent cytokine and chemokine production. In addition to its role as a PRR, NOD2 can participate in the regulation of intracellular signaling cascades [46,47].

We studied the role of NOD2 using a semi-allogeneic model of GVHD and found that splenic or bone marrow-derived DCs (BMDCs) from NOD2 deficient mice, given together with the transplanted cells, protected from aGVHD dependent mortality. Curiously, at early time-points (20 days post-BMT) no differences in clinical scores, bacteremia or histopathology were observed between animals receiving NOD2 KO or control DCs. At 40 days post-BMT however, protection was correlated with diminished bacteria translocation and histological alterations in all organs analyzed. These results indicate that absence of NOD2 protects from GVHD related mortality, but do not interfere with early disease, allowing the study of transplant related mortality independent of GVHD severity.

## MATERIALS AND METHODS

### Mice

C57BL/6 (B6, H-2^b^), F1 (C57BL/6 x BALB/c, H-2^bxd^) and NOD2KO (C57BL/6 background) mice were bred at the animal facility of the Brazilian National Cancer Institute (INCA, Rio de Janeiro, Brazil). NOD2KO were provided by the animal facility of the Department of Biochemistry and Immunology, School of Medicine of Ribeirão Preto, USP (Ribeirão Preto, Brazil). C57BL/6 mice were used as wild type controls. The Brazilian National Cancer Institute Ethics Committee for Animal Research approved all experimental research, under protocol #012/13.

### Bone Marrow Transplantation

F1 hosts received 950cGy and 24h post-irradiation were reconstituted with 5×10^6^ bone marrow (BM) cells from F1, B6 or NOD2KO as indicated. Depending on the indicated experimental settings, 5x10^6^ T cells either purified or from total splenocytes, together or not with 10^6^ B6 or NOD2KO bone marrow-derived dendritic cells (BMDCs) were injected into the tail vein of irradiated hosts.

### Cell Depletion and purification

Total splenocytes were incubated with biotin-conjugated antibodies against B220 (RA3-6B2), CD4 (GK1.5), CD8a (53-6.7) and CD49b (DX5) for lymphoid cells depletion, or with antibodies against CD11b (M1/70), CD11c (N418), I-A/I-E (M5/114.15.2), CD4 (GK1.5) and CD8a (53-6.7) (eBioscience, San Diego, CA) for myeloid and T cell elimination. Cells were washed and incubated with streptavidin-conjugated magnetic microbeads and negative selection was carried out in a MACS CS column as indicated by manufacturer (Miltenyi Biotec, Auburn, CA). T cells were purified from peripheral lymph node (LN) incubated with biotin-conjugated antibodies against B220 (RA3-6B2), Ly76 (TER-119), CD11b (M1/70), CD11c (N418), I-A/I-E (M5/114.15.2) and CD49b (DX5) (eBioscience). Cells were washed and incubated with Dynabeads Biotin Binder (Invitrogen, Oslo, Norway) following manufacturer instructions. T cells were >95% pure.

### Generation and activation of bone marrow-derived dendritic cells

BMDCs were generated from B6 wild type or NOD2KO mice as previously described [48]. For activation BMDCs were treated for 24 hours with 100 ng/mL of lipopolysaccharide (Sigma-Aldrich, St. Louis, MO), 2 μg/mL of total protein from heat-killed *Staphylococcus aureus* or 2 μg/mL of peptidoglycan from *S*. *aureus* and stained with antibodies against I-A/I-E (M5/114.15.2), CD80 (16-10A1), CD86 (GL1) and CD40 (3/23) (eBioscience, San Diego, CA). Data was acquired in FACSCalibur and analyzed with Cell Quest Pro (BD, Franklin Lakes, NJ) or Flowjo (Tree Star, Ashland, OR) software.

### Clinical GVHD assessment

GVHD score was modified from the literature [49,50] and performed based on 5 parameters: weight loss, fur texture, posture, activity and diarrhea. Each parameter receives a value from 0 to 2, according to severity. Total clinical score was obtained by adding individual parameters values. Humane endpoints were used. Transplanted animals, which reached global clinical score of 8 or activity score of 2, were sacrificed by an intraperitoneal administration of a lethal dose of ketamine (375 mg/kg) and xylazine (100mg/kg). The animals were monitored twice a week.

### Histopathologic GVHD

All samples were prepared for standard light microscopy examination. Scoring system was modified from the literature [51–54]. The following parameters were evaluated—Skin: inflammatory infiltration, fibrosis and loss of appendages, epidermal changes, ulceration; Liver: global parenchyma change (tumefaction/steatosis), portal space infiltration, parenchyma infiltration, parenchyma distress (necrosis/apoptosis); Colon: lamina propria infiltration, deeper layer infiltration, structural changes, damage extension. Each parameter receives a value from 0 to 2, according to severity.

### Regulatory T cell assessment

Dorsal skin samples were individually collected, sliced in small pieces and incubated for 30 minutes at 37°C (80–100 rpm) in HBSS solution containing 20 mM HEPES, 10% fetal bovine serum (FBS), 0.5 M EDTA and 0.1 mM dithiothreitol (DTT, Sigma-Aldrich) in agitation. Colon tissue samples were individually collected, washed extensively with a Ca^2+^ and Mg^2+^ free HBSS solution, longitudinally opened, sliced in small pieces and incubated, as the skin, but for 1 hour, at RT in agitation. The remaining tissue was collected and incubated in a HBSS solution containing 100 U/mL of type II collagenase, for an extra 1 h, at 37°C in agitation. Liver was perfused with 0,9% NaCl saline solution, sliced in small pieces and incubated in DMEM supplemented with 100U/mL of type II collagenase for 30 minutes at 37°C in a shaker (80–100 rpm). Lymphoid organs were macerated, cells were collected, washed in DMEM with 10% FCS. All samples were stained with anti-CD25 (PC61.5), FoxP3 (FJK-16) and CD4 (GK1.5) antibodies, acquired in FACSCalibur and analyzed with Cell Quest Pro (BD) or the Flowjo (Tree Star) software.

### Cytokine detection

Serum from transplanted mice were collected at indicated times. Luminex kits were purchased from BD Biosciences and Millipore (Millipore, Billerica, MA). The proceedings were carried out as specified by the manufacturers.

### LPS detection

Serum from transplanted mice were collected at indicated times and LPS measured using the LAL assay (LONZA, Walkersville, MD).

### Gene expression

BMDC total RNA was isolated and cDNA synthesis was performed using TRIzol (Life Technologies). qRT-PCR was done using TaqMan Gene Expression Assays (Applied Biosystems, Carlsbad, CA) for Arginase1 (Mm00475988_m1), Nitric Oxide Synthase 2 (Mm00440502_m1) and GAPDH (Mm99999915_g1) 20X on a 7500 Fast Real-time PCR system (Applied Biosystems, Carlsbad, CA).

### Morphologic analyses

Peripheral blood and bone marrow cells were prepared in a Cytospin2S (Shandon, Pittsburgh, PA). Slides were stained with H&E (Merck, Rio de Janeiro, Brazil) and analyzed by optical microscopy. Immature myeloid cells (myeloblasts, promyelocytes, myelocytes and metamyelocytes,) were grouped into a unique category called immature neutrophils.

### Statistical analysis

Data were analyzed using one-way or two-way ANOVA with Bonferroni post-test. Survival data were analyzed with log-rank test. Error bars represent Standard Deviation and * *p* < .05, ** p < .01 and *** p < .001. All the data were analyzed with Prism 5.0 (Graphpad, La Jolla, CA).

## RESULTS

### Absence of NOD2 in donor myeloid cells attenuates aGVHD

**Fig 1:**
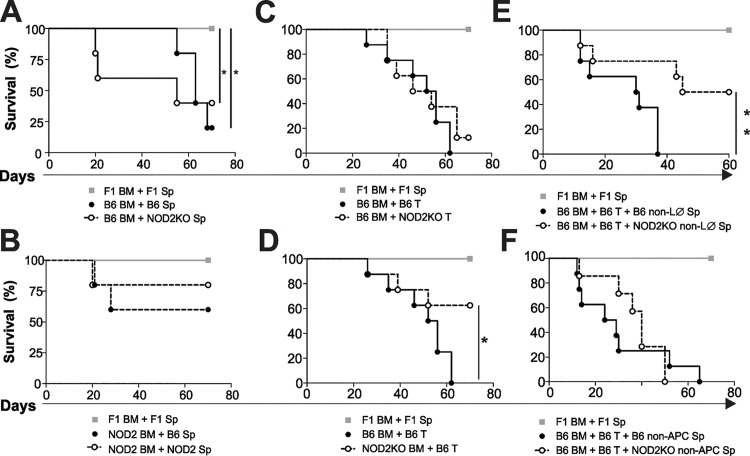
NOD2KO bone marrow myeloid cells protect mice from GVHD-related mortality. F1 (bxd) mice were lethally irradiated and received BM cells (5x10^6^) along with splenocytes (corrected to 5x10^6^ CD3^+^ cells) derived from F1, WT or NOD2KO as indicated (A-B). F1 mice (C57BL/6 x BALB/c) were lethally irradiated and received F1 WT BM cells and splenocytes as syngeneic control or **(C)** WT BM cells along with WT or NOD2KO purified T cells, **(D)** WT or NOD2KO BM cells along with WT purified T cells **(E)**, WT BM cells and purified T cells along with B6 WT or NOD2KO non-T, non-B and non-NK spleen cells (non-LØ), **(F)** WT BM cells and purified T cells along with WT or NOD2KO non -CD11b, -CD11c, -CD4 and -CD8 spleen cells (non-APC). The results are represented as percent of surviving animals. Pooled results from 2 experiments; n = 10 animals per group. Log-rank test for trend. *p<0,05 and **p<0,01.

To assess the role of NOD2 expression by donor hematopoietic cells in aGVHD post-BMT, lethally irradiated F1 (bXd) mice were transplanted with bone marrow cells and splenocytes from WT or NOD2KO mice (C57Bl/6 background). As shown in Fig. 1A, the absence of NOD2 in splenic cells did not affect survival rates. However, when BM cells were from NOD2 deficient mice, regardless the T cells origin, protection was observed (Fig. 1B). These results suggest that the absence of NOD2 expression in BM donor cells attenuates aGVHD.

To investigate which donor cell population is responsible for the protection in the absence of NOD2, lethally irradiated F1 mice were transplanted with purified T cells and BM cells from WT or NOD2KO mice. No difference was observed in the survival rates of mice receiving WT BM cells and either WT or NOD2KO T cells (Fig. 1C). Moreover, mice that received NOD2KO BM cells were protected from aGVHD, when compared to mice that received WT BM cells (Fig. 1D). Given that myeloid cells comprise more than 50% of the BM we asked whether the myeloid lineage was responsible for the protection. To assess this issue, lethally irradiated F1 mice were transplanted with WT BM cells plus WT purified T cells (allo) in the presence of splenocytes depleted of lymphoid cells, (T, B and NK cells) or myeloid cells (monocyte/macrophages, DCs and granulocytes) from WT or NOD2 KO mice. Protection was present in the absence of lymphoid but not of myeloid NOD2KO cells. (Fig. 1E and 1F).

### Acute GVHD protection is mediated by NOD2 KO BMDCs

**Fig 2:**
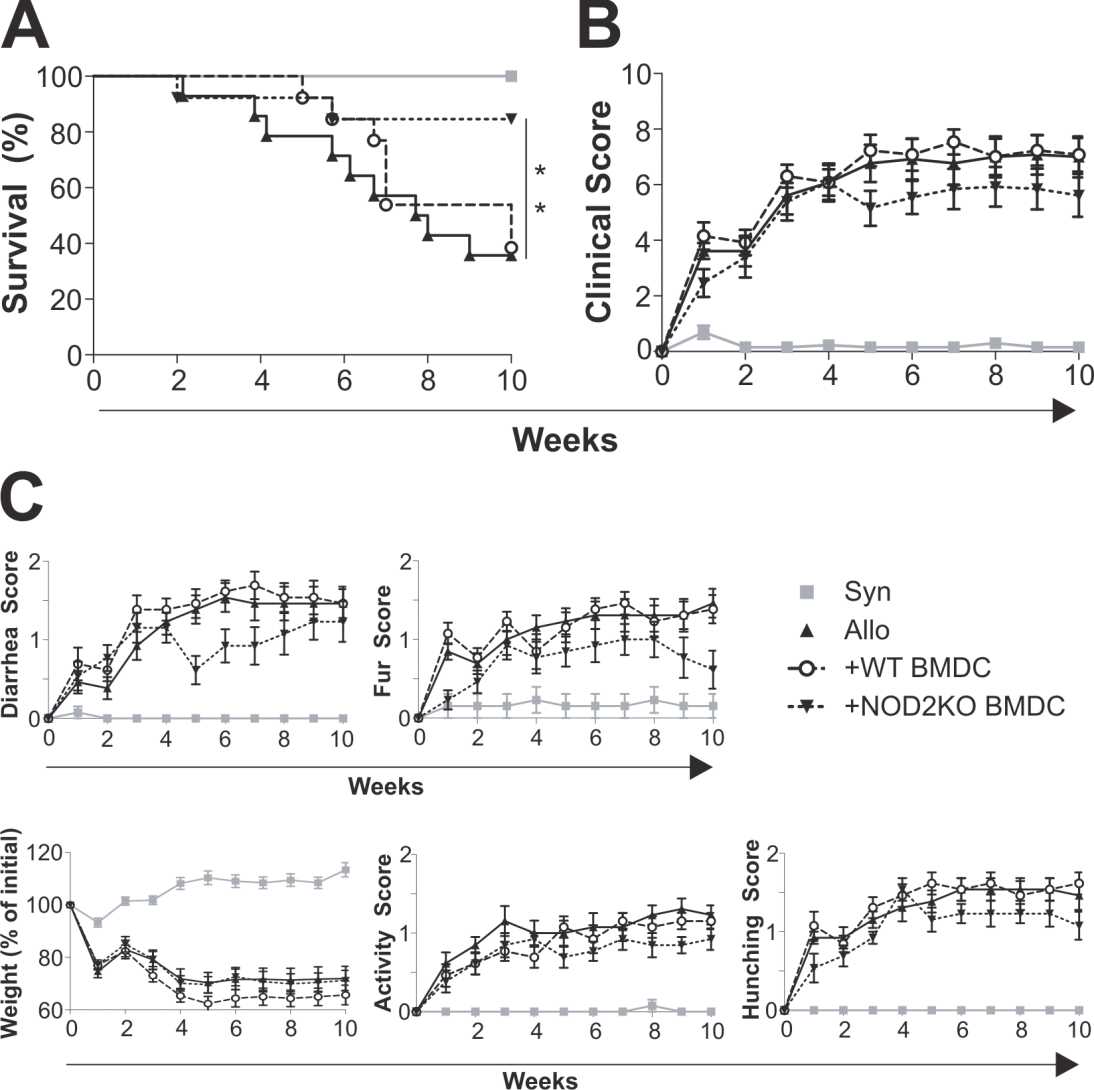
NOD2KO DCs impairs aGVHD mortality. F1 (bxd) mice were lethally irradiated and received F1 WT BM cells and splenocytes as syngeneic control or WT BM cells and purified T cells, along with WT or NOD2KO BMDCs. **(A)** Survival and **(B)** overall GVHD clinical scores are depicted. **(C)** Weight loss and individual scores for fur, diarrhea, activity and hunching. Log Rank test. *p<0.05. Pooled results from 3 experiments; n = 16 animals per group.

To check which population amongst the myeloid cells were responsible for the inhibitory effect, WT or NOD2KO bone marrow-derived dendritic cells (BMDCs) were generated and injected into GVHD semi-allo chimaeras. Mice that received NOD2KO BMDC had higher survival rate (>80%), assessed 10 weeks post-BMT, when compared with transplanted mice that received WT BMDC or nothing (<40%) (Fig. 2A). Surprisingly, when clinical score was considered, no significant difference was observed in the group protected from death with NOD2KO BMDC when compared to allo or WT BMDC groups (Fig. 2B and 2C). Thus, NOD2KO BMDC can protect mice from aGVHD induced death, although there is apparently no protection when clinical signs were considered.

### NOD2KO BMDC protects colon, liver and skin in a progressive fashion

**Fig 3:**
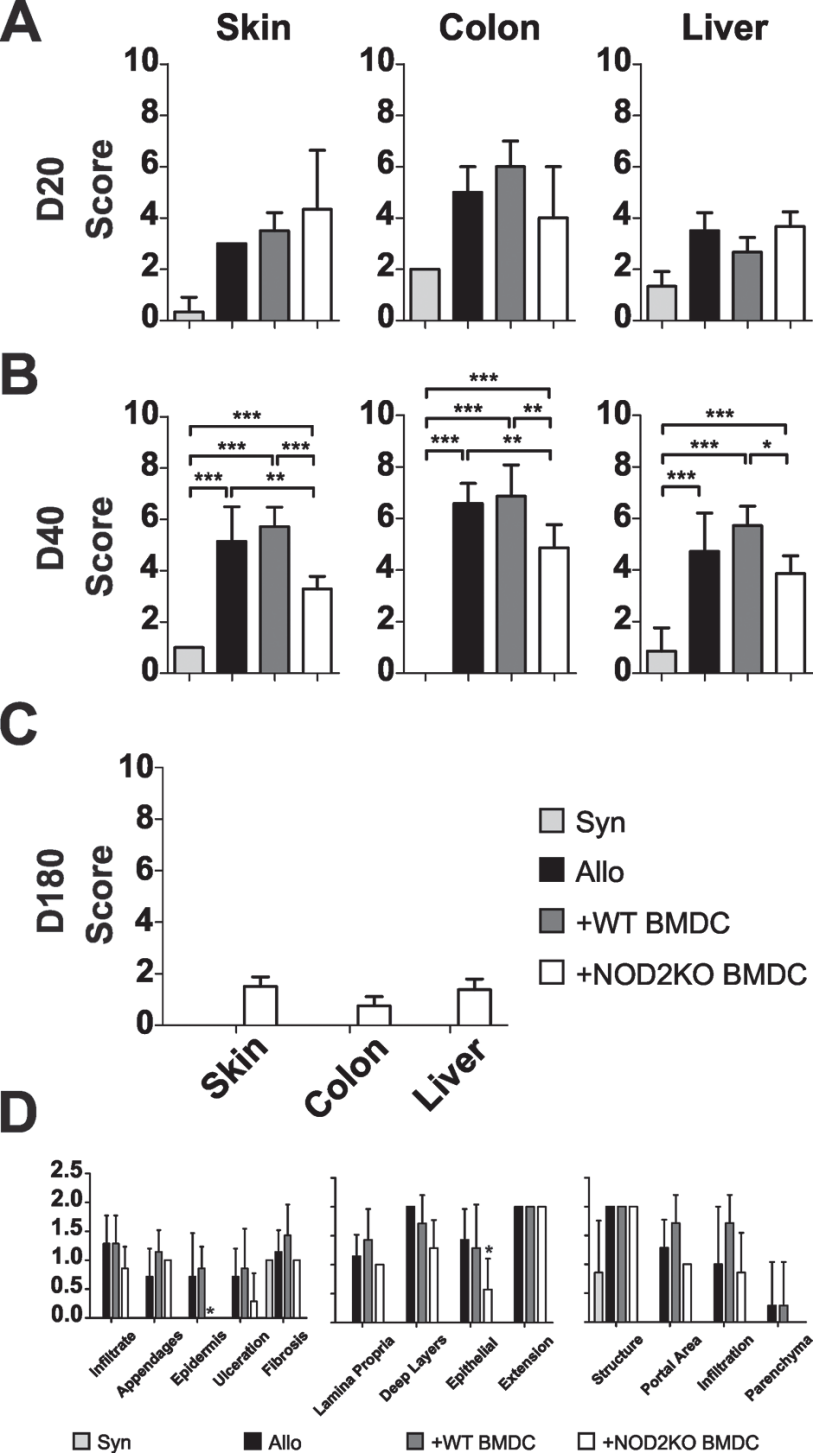
Mice that received NOD2KO BMDC developed histopathological progressive protection in skin, colon and liver. F1 (bxd) mice were lethally irradiated and received F1 WT BM and splenocytes as syngeneic control or B6 WT BM cells and B6 WT purified T cells, along with B6 WT or B6 NOD2KO BMDCs. Histopathology score of colon, liver and skin of animals after **(A)** 20, **(B)** 40 and **(C)** 180 days. **(D)** Parametric score of skin, colon and liver histopathology. One-way ANOVA with Bonferroni. *p<0.05, **p<0.01 and ***p<0.001. Pooled results from 3 experiments; n = 15 animals per group.

As NOD2KO BMDCs were able to protect transplanted mice from aGVHD lethality, but not from clinical disease, histopathological examination was performed. Allo transplanted mice that received either WT or NOD2KO BMDC showed similar histopathological scores in skin, colon and liver 20 days after transplantation (Fig. 3A). However, by day 40, significant lower scores were found. In contrast to mice in the positive control groups (allo and WT BMDC), which were all deceased by day 180, mice that received NOD2KO BMDC, remained alive, still with minimum histopathological signs of disease (Fig. 3B and 3C). The histopathological score is comprised by parameters related to tissue health and cellular infiltrate. To verify whether the histopathological protection is linked to any specific parameter, each parameter was individually evaluated at day 40 after transplantation. Mice that received NOD2KO BMDC showed increased epithelial protection as evidenced in skin and colon when compared with mice that received WT BMDC or nothing (Fig. 3D and S1 Fig.). Thus histopathological protection mediated by NOD2 KO BMDC, although observed in all tissues analyzed, was more pronounced in epithelial tissues, where progressive protection, with time, tend to reach the scores found in the syngeneic group (S1 Fig.).

### GVHD suppression was not due to different expression of molecules involved neither in antigen presentation nor to MDSC or Treg suppression

**Fig 4:**
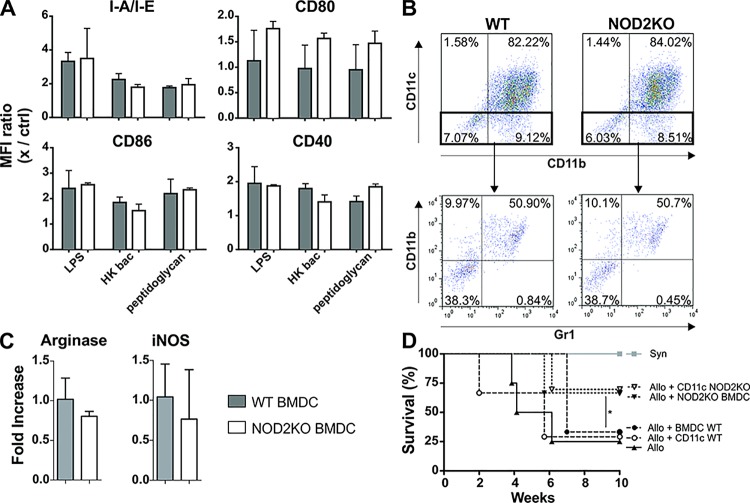
NOD2KO BMDC express lower levels of MHC-II when activated. **(A)** WT or NOD2KO BMDC, generated as indicated in material and methods, were treated with LPS, heat-killed bacteria or peptidoglycan and stained for evaluation of membrane expression of I-A/I-E, CD80, CD86 and CD40. One-way ANOVA with Bonferroni post-test, ***p<0.001. Pooled results from 2 experiments; n = 14 animals per group. **(B)** BMDC from WT and NOD2KO mice were stained for CD11b, CD11c and Gr1. **(C)** Real-time quantitative PCR for Arginase1 and iNOS in WT and NOD2KO BMDC. **(D)** F1 mice were lethally irradiated and received F1 WT BM cells and splenocytes as syngeneic control or WT BM cells and WT purified T cells, along with WT or NOD2KO BMDCs or purified CD11c cells from WT or NOD2KO. *p<0.05. Pooled results from 2 experiments; n = 10 animals per group.

To verify whether WT and NOD2KO BMDC activation and/or differentiation status differs we evaluated the surface expression of activation/lineage markers and co-stimulatory molecules in steady-state and activated BMDC. CD11c^+^ NOD2 KO and WT BMDC equally up-regulated CD80, CD86, CD40, CD83 and MHC-II (I-A/I-E) upon stimulation with either lipopolysaccharide (LPS), heat-killed bacteria (HK bac) or peptidoglycan (PEPG) (Fig. 4A). This data is correlated to the allo-stimulatory capacity of NOD2KO BMDC, which is similar to WT cells, as observed in a mixed leukocyte reaction (MLR) *in vitro* (data not shown). BMDCs are a heterogeneous population, where the majority of the cells were CD11c^+^/CD11b^+^ (Fig. 4B). The CD11c^-^ cells were CD11b single-positive (monocytic cells) [55] or CD11b/Gr1 double positive cells, a phenotype compatible with granulocytes, inflammatory monocytes or myeloid-derived suppressor cells (MDSC) [56–58]. Since MDSC regulate aGVHD pathogenesis [57], we evaluated arginase and iNOS expression, which are characteristics of MDSC when co-expressed [57]. Both WT and NOD2KO BMDC express the same levels of arginase and iNOS (Fig. 4C), suggesting that there are no differences regarding MDSC activity.

To verify whether the CD11c+ population mediated the protection, we purified the CD11c^+^ fraction and co-infused with BM and splenocytes. In fact, protection mediated by NOD2KO BMDC was due to CD11c cells (Fig. 4D). On the other hand, CD11c negative BMDC from NOD2KO mice did not protect from aGVHD (data not shown).

**Fig 5:**
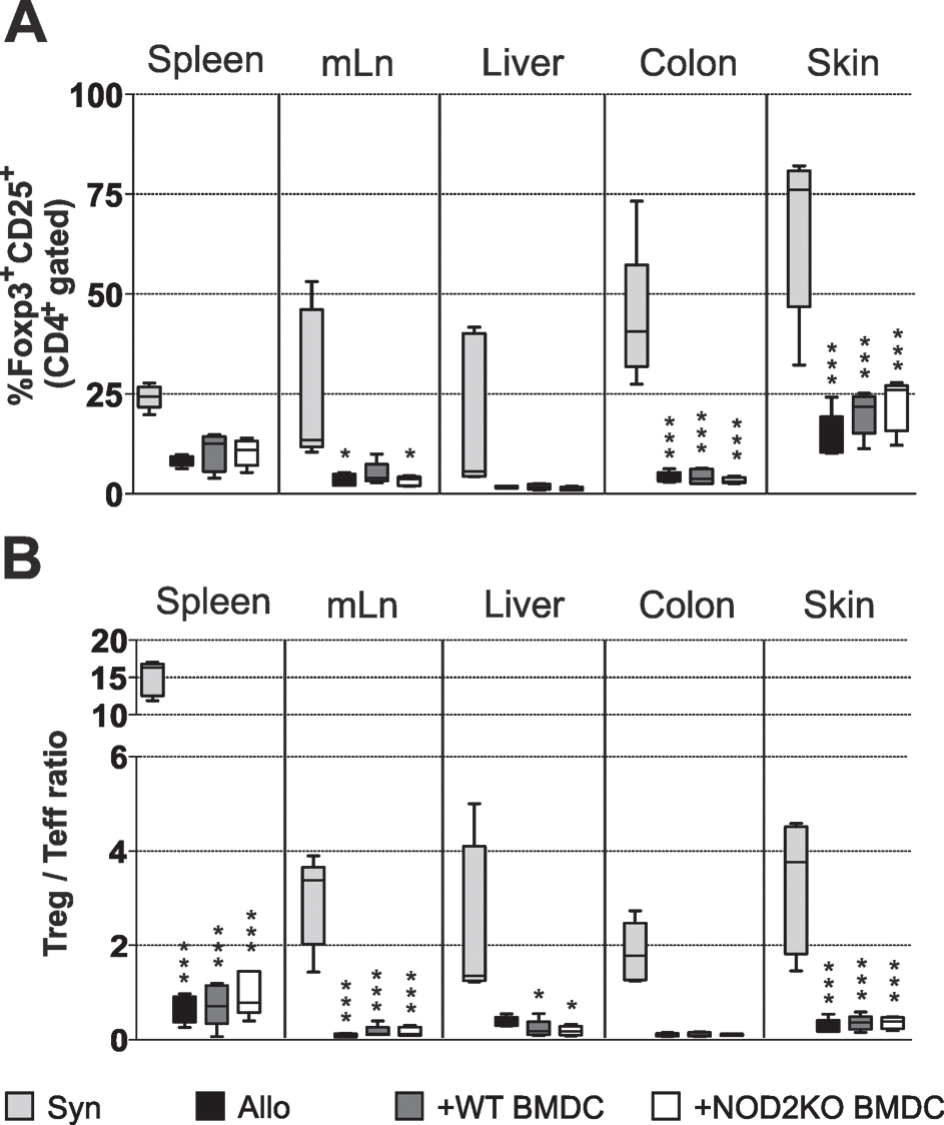
Mice that received WT or NOD2KO BMDC have the same frequency of Treg cells. F1 (bxd) mice were lethally irradiated and received F1 WT BM cells and splenocytes as syngeneic control or WT BM cells and WT purified T cells, along with WT or NOD2KO BMDC. After 21 days, spleen, mesenteric lymph nodes, colon, liver and skin of transplanted F1 animals were stained for CD4, CD25 and Foxp3 for the evaluation of the **(A)** percentage of Treg cells (CD4^+^CD25^+^Foxp3^+^) and **(B)** the Treg:Teff ratio. One-way ANOVA with Bonferroni post-test, *p<0.05 and ***p<0.001. One experiment representative of 2; n = 5 mice per group.

CD4^+^CD25^+^FoxP3^+^ regulatory T cells (Tregs) were shown to be protective in aGVHD and induced by tolerogenic DCs [59]. To verify whether the protection was dependent on Treg, 21 days after transplantation, secondary lymphoid organs, liver, colon and skin were assessed for Treg frequencies. Transplanted mice which received syngenic T cells displayed higher Treg numbers and Treg/effector T cell ratios in all organs analyzed when compared to mice which received allogenic T cells and therefore are suffering from aGVHD (Fig. 5A and 5B) [60]. Mice transplanted with WT BM and T cells in the presence or absence of WT BMDC or NOD2 KO BMDC showed the same Treg absolute numbers, frequency and Treg/Teff ratio (Fig. 5A and 5B) [60]. The absence of differences in Treg frequencies between the various groups tested, suggests that Tregs are not involved in the mechanism by which NOD2 KO BMDC inhibits aGVHD related mortality.

### NOD2KO BMDC protection from aGVHD mortality correlates with epithelial barrier maintenance

**Fig 6:**
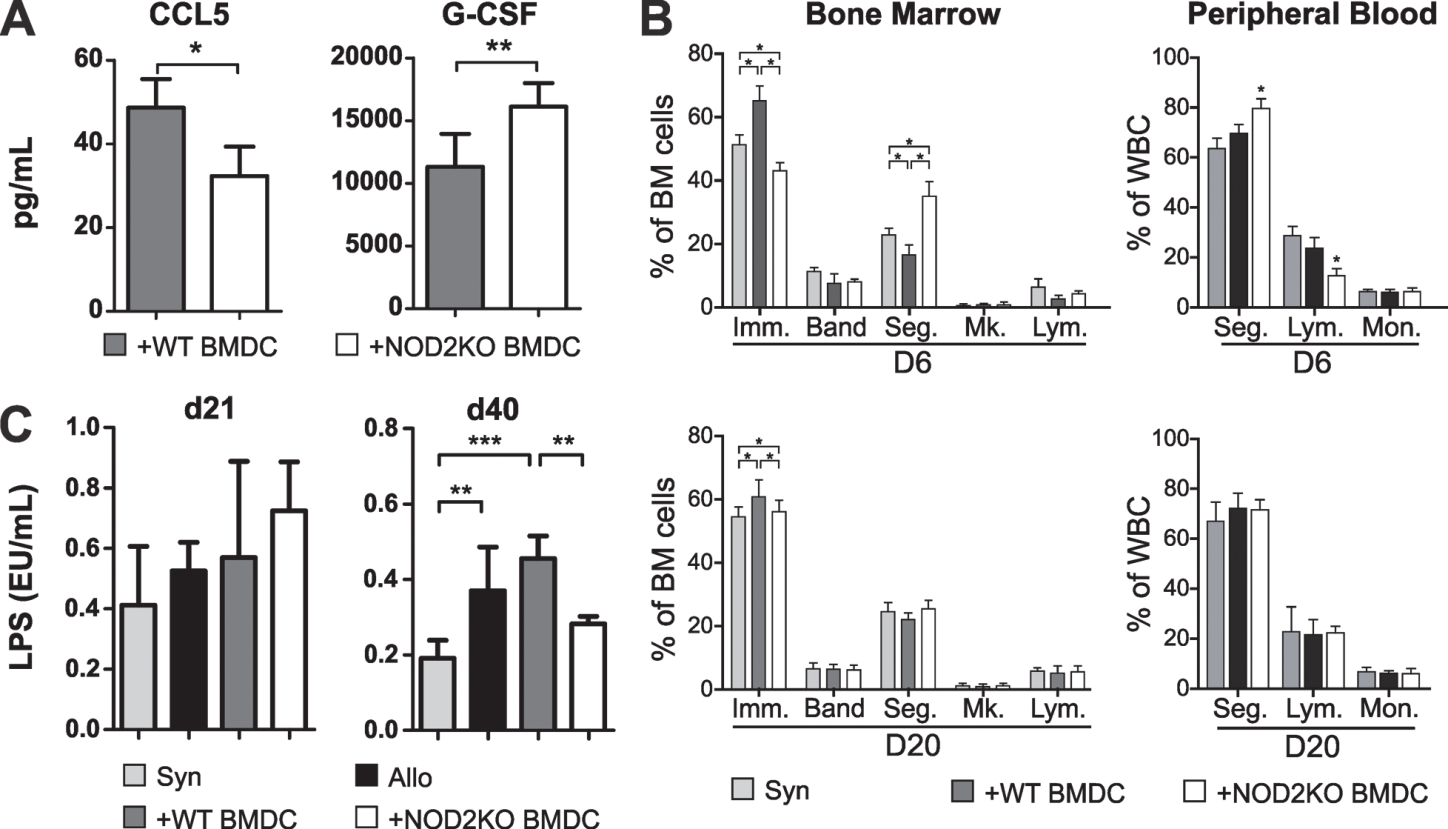
Mice that received WT or NOD2KO BMDC had lower levels of CCL5 and LPS and higher levels of G-CSF. F1 (bxd) mice were lethally irradiated and received F1 WT BM cells and splenocytes as syngeneic control or WT BM cells and purified T cells, along with WT or B6 NOD2KO BMDC. Serum of transplanted animals was collected 21 days post transplantation. **(A)** CCL5 and G-CSF were analyzed by multiplex ELISA. **(B)** Bone marrow and blood from transplanted animals were collected on day 6 and 20, cytocentrifuged and stained with H&E for differential cell counts, **(C)** LPS from individual mice was quantified on days 21 and 40 after transplantation using the LAL assay. T test for A and One-way ANOVA with Bonferroni post test for B. *p<0.05, **p<0.01 and ***p<0.001. Pooled results from 2 experiments; n = 10 mice per group.

Since our results indicated that NOD2KO BMDC is implicated in epithelial injury we evaluated parameters of systemic inflammation. We checked the serum cytokine profile of transplanted mice. Chimaeras that received NOD2KO BMDC showed lower levels of CCL5 and higher levels of granulocyte-colony stimulating factor (G-CSF) when compared to control chimaeras (Fig. 6A). Levels of TNF-α, IFN-γ, IL-2, IL-4, IL-6, IL-10, IL-17 IL-12p40 and IL-12p70, were similar in both groups, although a statistically non-significant increase in CXCL1, TNFα, IL12p40 and IFNγ was observed (S2 Fig.) suggesting overall increase in inflammatory activity. The increased G-CSF levels encountered 20 days post-BMT correlated with increased granulopoiesis with lower frequencies of immature neutrophils and higher frequencies of segmented neutrophils in the BM when compared to mice that received WT BMDC (Fig. 6B). At day 20, immature BM neutrophils numbers were increased, but the percentage of mature cells was not (Fig. 6B). To assess bacterial translocation due to epithelial barrier damaged, we measured serum lipopolysaccharide (LPS) and found no difference between the experimental groups 20 days post-BMT (Fig. 6C). However, at day 40 after transplantation, mice that received NOD2KO BMDC displayed similar LPS levels encountered in healthy animals, significantly lower than the levels observed in animals that received allogenic cells with or without WT BMDCs (Fig. 6C). Our data suggests that NOD2KO BMDC at early time points induces systemic G-CSF increase, which correlates with an improved neutropoiesis. Moreover, at later time points, bacterial translocation was diminished in NOD2KO BMDC transplanted mice, once more indicating progressive protection of epithelial tissues that is associated with protection from death.

## DISCUSSION

Mammalian NLR proteins are believed to function as intracellular pattern recognition receptors recognizing muramyl dipeptides from bacterial cell wall [61,62]. NOD1 and NOD2 had been shown to be involved in bacterial [63] as well as in other intracellular infections [47,64]. Particularly, the *nod2* gene had been described as a positive modulatory element in a model of PG-induced arthritis [65] and autoimmune liver injury [66]. Moreover, for Crohn’s Disease, *nod2* polymorphism [67] had been shown as a risk factor, increasing in 15–40 fold the chance of developing disease [68].

The importance of the intestines as target organs in GVHD had inspired studies addressing the role of *nod2* gene polymorphism in the outcome of HSCT. Although, the results in human patients are somewhat conflicting [35–38,40–43] most studies indicate that NOD2 acts as a negative regulator of inflammation, since SNPs 8, 12, 13 and other similar *nod2* variants, in both donor and recipient correlate with acute and chronic GVHD increased severity, non-relapse mortality, epithelial disease and overall survival [43]. HSCT outcome due to *nod2* variants depends on primary disease and conditioning regimen, specially the usage of antibiotics and T cell depletion strategies [43].

Injection of NOD2KO BMDC with the transplant protects animals from death, a result somewhat unexpected given the results published by Penack et al. [41] where, in a different experimental setting, deficiency of NOD2 in the hematopoietic tissue of recipient mice increased aGVHD mortality and severity. Our results are corroborated by the findings from one human study, which showed complete protection from severe aGVHD when the donor carried variants forms of NOD2 associated with Crohn’s disease [35]. Although in our settings, the only cellular population with impaired NOD2 expression was BMDCs, a heterogeneous population comprising mainly CD11c^+^ cells, but also CD11b^+^ cells and Gr1^+^/CD11b^+^ cells, the latter being a phenotypic characteristic of some MDSC populations [57]. GVHD is inhibited by MDSC due to L-arginine depletion through iNOS and arginase-I concomitant expression, therefore decreasing T cell activity [57]. We found no differences between WT and NOD2KO BMDC regarding iNOS and arginase-I expression. DCs are known to instruct the differentiation of inducible regulatory T cells (iTregs), especially in epithelial tissues [69]. However, accumulation of regulatory T cells in GVHD target organs was similar between all experimental groups. In the present experimental model, protection was observed only when survival and specific histopathological analysis were considered. Although present in all tissues analyzed after 40 days, protection was markedly related to epithelial tissues, as evidenced in gut and skin histopathology. Mice that received WT BMDC presented epidermis thickening in skin and epithelial hyperplasia and gut disorganization, which were ameliorated by co-transfusing NOD2KO BMDC. Interestingly, tissue protection was progressive as observed 20, 40 and 180 days post-BMT indicating that the effect of NOD2KO BMDC endures. One possible explanation is that NOD2KO BMDCs have a role in hematopoiesis with long lasting consequences for GVHD as a consequence of a discrete, although significant faster hematologic recovery. We observed increased G-CSF levels in mice receiving NOD2 KO BMDCs. This suggests that KO BMDCs, are actively promoting granulopoiesis through a yet unknown mechanism. Neutrophils possess as primary function the ability to control microorganisms, capacity that is optimized by G-CSF. As part of GVHD pathogenesis, translocation of microorganisms, especially bacteria through damaged epithelia are related to increased inflammatory response, T cell alloresponse and therefore, disease progression. We observed that, protected mice presented decreased amounts of circulating LPS, which is an indicative of bacteremia especially after 40 days post BMT. Possibly, NOD2KO BMDC protection is exerted by microbicidal mechanisms of neutrophils, which, in turn protect tissues from inflammatory damage, especially those that are colonized by commensal bacteria, such as epithelial surfaces. In additon, NOD2 in myeloid cells might participate on the selection of commensal flora, as it occurs in epithelial Paneth cells [70], and this determines survival as an endpoint, adding a risk factor for mortality in aGVHD [71].

Although the precise mechanism by which NOD2 in donor BMDC modulates GVHD needs further elucidation, the present findings raise two important questions: one regards the use of histopathology as a follow up tool considering different survival rates in the presence of similar cellular infiltration observed in the liver and intestines and similar clinical signs between protected and non-protected mice. The second question regards the mechanism of suppression, which is clear by the low histopathological scores at later time points implicating establishment of long lasting tolerance.

In conclusion, NOD2 deficient BMDC can protect from lethal aGVHD. On the short term, survival increase does not correlate with clinical nor general histopathology protection but with a faster engraftment and specific epithelial protection. On the medium/long term, decreased bacteremia evidenced by lower levels of circulating LPS and tissue recovery might explain survival protection. These findings suggest that GVHD induced death is an indirect phenomena, not strictly related to disease severity or general tissue damage and open a new possibility for understanding the discrepancies between pathology and mortality related to aGVHD.

## Supporting Information

S1 Fig Histopathological analysis of skin, colon and liver show a discrete protection by NOD2KO BMDC on day 40 post transplant. F1 (bxd) mice were lethally irradiated and received and received F1 WT BM and splenocytes as syngeneic control or WT BM and WT purified T cells, along with WT or NOD2KO BMDC. 40 days after transplantation colon, liver and skin were processed for histological examination and H&E stained. Representative micrographs are shown. 400X magnification.
(PDF)

S2 Fig Serum cytokine profile from transplanted animals. F1 (bxd) mice were lethally irradiated and received F1 WT BM and splenocytes as syngeneic control or WT BM cells and WT purified T cells, along with B6 WT or B6 NOD2KO BMDCs. Sera from transplanted animals were collected 21 days post transplantation. Cytokines were analyzed by using multiplex ELISA. Pooled results from 2 experiments; n = 10 mice per group.
(PDF)
