# Supplementary figures and images for: Disease Severity and Mortality Can Be Independently Regulated in a Mouse Model of Experimental Graft versus Host Disease
Source: PLoS One. 2015 Feb 2;10(2):e0118079. doi: 10.1371/journal.pone.0118079 (PMC4313938; doi:10.1371/journal.pone.0118079)

Figure S1

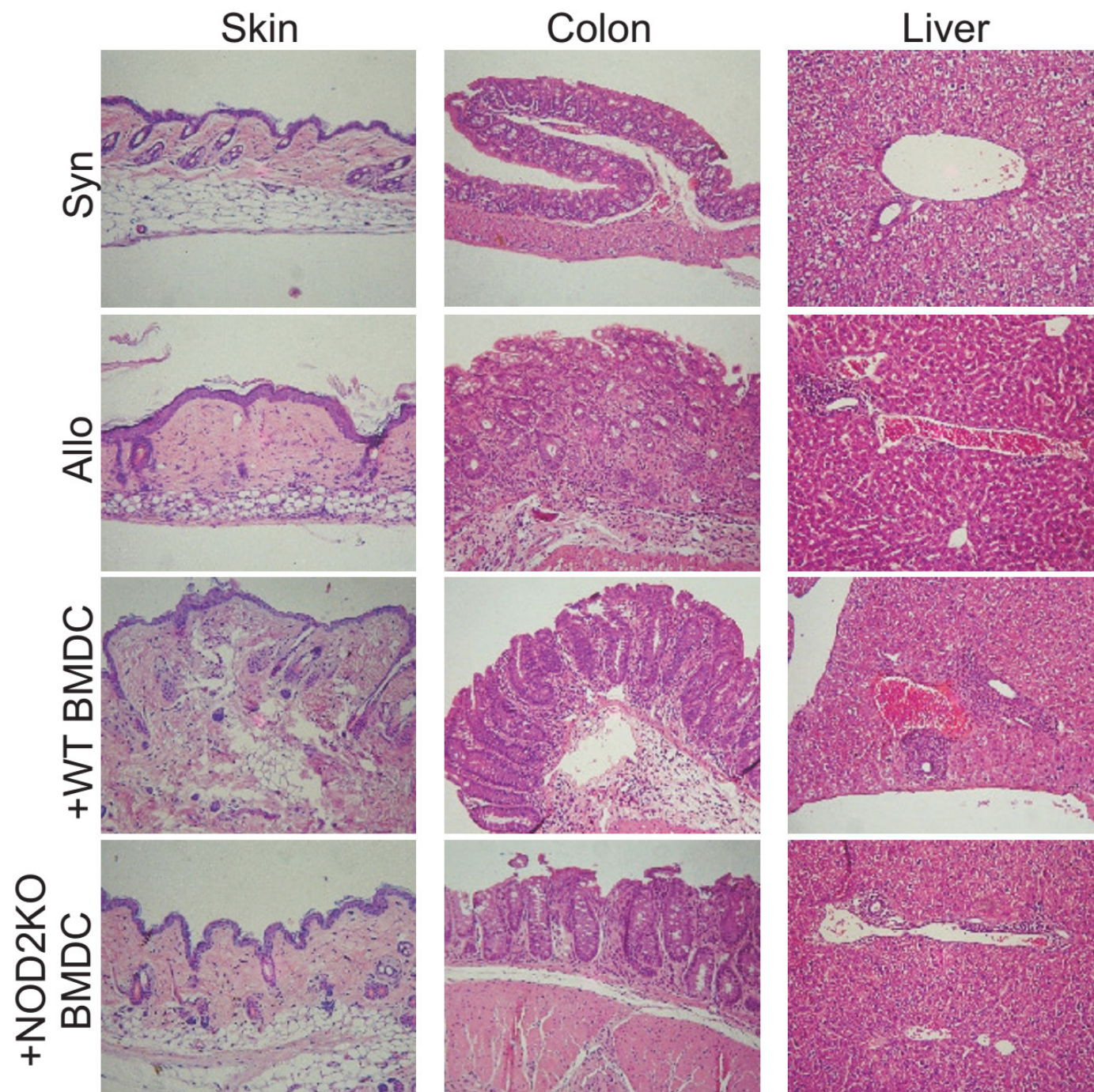

Supplement: S1 Fig — F1 (bxd) mice were lethally irradiated and received and received F1 WT BM and splenocytes as syngeneic control or WT BM and WT purified T cells, along with WT or NOD2KO BMDC. 40 days after transplantation colon, liver and skin were processed for histological examination and H&E stained. Representative micrographs are shown. 400X magnification. (PDF) [file pone.0118079.s001.pdf]

Figure S2

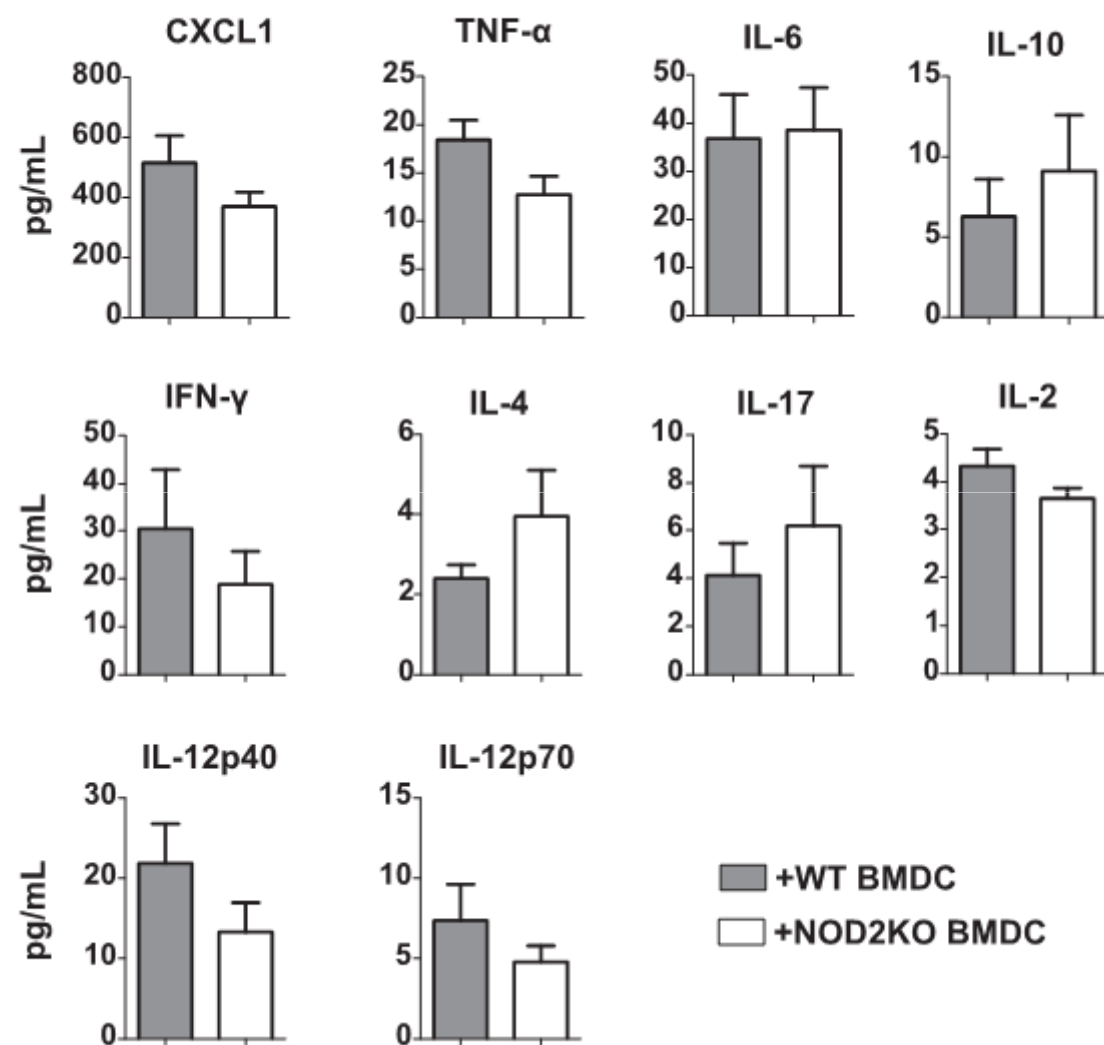

Supplement: S2 Fig — F1 (bxd) mice were lethally irradiated and received F1 WT BM and splenocytes as syngeneic control or WT BM cells and WT purified T cells, along with B6 WT or B6 NOD2KO BMDCs. Sera from transplanted animals were collected 21 days post transplantation. Cytokines were analyzed by using multiplex ELISA. Pooled results from 2 experiments; n = 10 mice per group. (PDF) [file pone.0118079.s002.pdf]
